# “You kind of blame it on the alcohol, but. . .”: A discourse analysis of alcohol use and sexual consent among young men in Vancouver, Canada

**DOI:** 10.1177/13634593231214942

**Published:** 2023-12-14

**Authors:** Trevor Goodyear, John L Oliffe, Hannah Kia, Emily K Jenkins, Rod Knight

**Affiliations:** University of British Columbia, Canada; British Columbia Centre on Substance Use, Canada; Wellstream: The Canadian Centre for Innovation in Child and Youth Mental Health and Substance Use, Canada; University of British Columbia, Canada; University of Melbourne, Australia; University of British Columbia, Canada; University of British Columbia, Canada; Wellstream: The Canadian Centre for Innovation in Child and Youth Mental Health and Substance Use, Canada; British Columbia Centre on Substance Use, Canada; Université de Montréal, Canada; Centre de Recherche en Santé Publique (CReSP), Canada

**Keywords:** alcohol, gender-based sexual violence, men, sex, sexual consent, youth

## Abstract

There is growing awareness about issues of sexual consent, especially in autonomy-compromising or “non-ideal” contexts, including sex involving alcohol. Understanding the conditions needed for consensual sex to occur in this emergent milieu is critically important, especially for young men (ages 18–30 years) who normatively combine drinking alcohol with sex and are most often perpetrators of sexual violence. This study offers a discourse analysis of young men’s alcohol use and sexual consent. Data are drawn from qualitative interviews with 76 young men (including gay, bisexual, queer, and straight men) in Vancouver, Canada, from 2018 to 2021. Informed by Kukla’s non-ideal theory of sexual consent and critical and inclusive masculinities, this analysis identified three discursive frames: *careful connections, watering it down*, and *blurred lines*. In *careful connections* young men discussed their efforts to actively promote sexual and decisional autonomy for themselves and their sexual partners when drinking. Yet, in *watering it down* young men invoked discourses of disinhibition, deflection, and denial to normalize alcohol use as being somewhat excusatory for sexual violence, downplaying the role and responsibility of men. Lastly, men operationalized *blurred lines* through a continuum of consent and of “meeting (masculine) expectations” when discussing sexual violence and victimization while intoxicated. Together, these discursive frames provide insights into the gendered nature of sexual violence and the extent to which idealized notions of sexual consent play out in the everyday lives of young men who use alcohol with sex. Findings hold philosophical and pragmatic implications for contemporary efforts to scaffold sexual consent.

## Introduction

Drinking alcohol with sex is common among young adults (ages 18–30 years) and has been the focus of much research in the health and social sciences (Cho and Yang, 2023; George, 2019; Wagenaar et al., 2018), such as in recent gender-based analyses (Hunt et al., 2021; Wilkinson and Wilkinson, 2020). The use of alcohol with sex can ease social and sexual encounters by facilitating connection and reducing inhibitions, including among gay, bisexual, transgender, and queer (GBTQ) men and others with non-(hetero)normative sexual relations and practices (Holmes et al., 2021; Goodyear et al., 2023). However, drinking with sex is also implicated in adverse sexual health experiences and outcomes. One meta-analysis of 50 studies identified associations between young people’s alcohol use and numerous “risky sexual behaviours,” including early sexual initiation, inconsistent condom use, and having multiple sexual partners, with risks varying by gender, sexual orientation, and other contextual factors (Cho and Yang, 2023). Critically, work in this sphere has also established links between alcohol use and the violation of sexual consent, including sexual aggression and violence, most often (yet not exclusively) perpetrated by men (Abbey, 2011; Norona et al., 2021).

In many contemporary contexts, including Canada, where the current study is set, young men (and others) are increasingly navigating issues of alcohol use and sexual consent in an evolving social milieu featuring the #MeToo movement and its empowering of sexual assault survivors to be heard and recognized, and for perpetrators of sexual violence to be held accountable (Boyle and Rogers, 2020; Gaspar et al., 2021; Jaffe et al., 2021). #MeToo has indeed sparked widespread re-examinations of sexual consent, including in potentially compromising and non-ideal situations, such as when using alcohol with sex (i.e. before, during, after). This is significant insofar as, while past work has suggested sexual assaults involving alcohol are less likely to be acknowledged, survivors’ perceptions of these experiences are neither universal nor static and are heavily shaped by social context (Jaffe et al., 2021). Of note, Jaffe et al.’s (2021) study of perceptions of sexual assault since the #MeToo movement began found that trends in average acknowledgement have changed over time and are also influenced by sexual assault characteristics, including degree of intoxication. Evolving discourses around sexual victimization in the era of #MeToo have heretofore centered the experiences of women and girls, though there has been growing interest in examining the movement’s implications for men and queer communities (Gaspar et al., 2021). Still, more work is needed to uncover how young men—across diverse groups (e.g. cisgender, heterosexual, and GBTQ men) and contexts (e.g. with sex involving alcohol) —are making sense of sexual consent discourses alongside our shifting socio-cultural landscape, including in connection with evolving gender norms and sexual scripts.

While young people of all genders use alcohol with sex, young men are most often accused, charged, and convicted as the perpetrators of sexual violence, including and perhaps especially when alcohol is involved (Carline et al., 2017; Garcia et al., 2019). However, it is critical to emphasize that sexual violence (encompassing sexual harassment, violation, and assault) is also known to occur and be under-reported among men (Dame et al., 2020; Depraetere et al., 2018), with disproportionately high rates among younger men and GBTQ men (Dame et al., 2020; Rothman et al., 2011). Sociological theories link men’s conquest, sexual prowess and/or aggressions to hypermasculine cultures (Connell, 2005; Murnen, 2015) wherein the pursuit of status, toughness, and antifemininity are foundational to many young men’s masculine identities. In this context, Weiss (2010a) theorizes that men who are victims to sexual violence contradict hegemonic definitions of men’s sexuality that require them to be sexually potent, dominant, and in control; and that men who deny or resist another’s sexual advances can be scrutinized as violating codes of male (hyper)sexuality. These arguments are substantiated by Weiss’s (2010a, 2010b) findings about how men with diverse sexual orientations seek to reassert masculinity following sexual victimization, including by blaming alcohol consumption for their loss of control and vulnerability. Gender relations as socially constructed practices have, across a continuum of social contexts, shaped the ways in which drinking can influence normative understandings and parameters for sexual consent and victimization. To this point, research with cisgender, heterosexual young adults reported that intoxicated sex can involve a reconfiguration of what are considered to be acceptable practices, in ways that are shaped by gender norms and cultural sexual scripts (Hunt et al., 2021).

Social sciences and humanities scholarship has sought to identify the conditions needed for consensual sex to occur, including when autonomy is potentially compromised by alcohol use. As Kukla (2021) argues in “*A non-ideal theory of sexual consent*,” the normative portrayal of sexual consent emphasizes the need for full decisional autonomy to legitimately consent to sex, significantly limiting opportunities for consensual sex to occur in the context of alcohol use. Indeed, key elements attributed to consensual sex tend to feature willingness (to have sex), communication (verbal/nonverbal cues), and interpretation (of the other’s communication and willingness; Beres, 2007; Kukla, 2021; Muehlenhard et al., 2016)—and, these approaches highlight (and rely on) the capacity for full decisional autonomy. Others have argued that understandings of sexual consent need to more fulsomely account for how autonomy and agency occur on shifting temporal continua (Whittington, 2021), including in relation to factors such as alcohol intoxication and evolving gender and sexuality norms, as has been surfaced in recent work with GBTQ men (Gaspar et al., 2021). Thus, accounting for how autonomy can be compromised by limitations in decisional capacity, while explicitly connecting consent dynamics to other ingredients needed for “ethical” sex, including the ability to exit a situation, trust, safety, and broader social supports, are key to evaluating sexual consent (Kukla, 2021).

Young men’s alcohol use and negotiation of sexual consent are shaped by masculine and feminine ideals. Dominant discourses of masculinity prize sexual prowess as a characteristic of successful masculinity—evidenced by conquest—and the pursuit of, and by, potential partners to validate and affirm that masculine capital (Anderson, 2009; Connell, 2005). The antithesis of this can be conceived as rejection and contested (masculine) identities within young men’s dating and hook-up cultures, with the longer-term risk of men not attracting a partner at all. In this context, alcohol as a potential connector can heighten hopes, loosen uncertainties and be the potentiator of (and disclaimer for) risk and young men’s risk-taking (Holmes et al., 2021; Iwamoto et al., 2011). As such, the performativity of drinking and the perceived prizes that can accompany such practices are understood as normative among young men (Hunt and Antin, 2019; Iwamoto et al., 2011; Petersson and Plantin, 2019). That is, by drinking, inhibitions are reduced, and the utopia of connectedness otherwise lost to everyday shyness and the often-awkward formative years may be seen as being more attainable. There are, however, strong countering discourses of masculinity whereby sex is not (only) about such hedonistic conquest-based masculinities but rather ensuring care of and mutual pleasure for men’s sexual partner(s) (Hunt et al., 2021; Wilkinson and Wilkinson, 2020). Protection of partners similarly counts as a dominant discourse of masculinity, while intoxication can be used to explain momentary lapses, with alcohol as a justification for such deviations as state-based rather than reflective of masculine shortcomings (Carline et al., 2017; Hunt et al., 2021). Contemporary masculine discourses inclusive of the practices above have been shaped by #MeToo (Gaspar et al., 2021; Jaffe et al., 2021), calling into question alcohol use and sexual consent in the retrospective amid forever changed terrain. Yet despite the eruption of the #MeToo movement globally, we know surprisingly little about how diverse young men are navigating sexual consent within this evolving social milieu and in non-ideal contexts, such as sex involving alcohol. Therefore, our aim in the present study is to identify young men’s discourses of alcohol use and sexual consent.

## Methods

### Positionality and theoretical perspectives

We approached this study as an interdisciplinary research team with training in nursing, public health, social work, and sociology. The lead author is a young cisgender queer man who, through his nursing practice and personal and community experiences, had developed some familiarity with interconnections between alcohol use, sex, and consent. Our broader authorship team includes men and women of diverse sexual identities (straight, bisexual, and gay) who are invested in researching and promoting young people’s community care practices, including in the context of sexual consent, with much of our work focused on advancing equity with sexual and gender minorities. For this study, we drew on Kukla’s (2021) non-ideal theory of sexual consent alongside critical and inclusive analyses of masculinities (Anderson, 2009; Connell, 2005). Following Connell (2005), we define masculinities as social and cultural structures that influence men’s (and others’) identities, practices, and relations. These effects of masculinity, including masculine hegemony, are known to be discursively (re-)produced and contested amid men’s everyday lives (Anderson, 2009; Connell, 2005), including in contexts of alcohol use, sex, and consent. In leveraging a critical, inclusive masculinities framework with Kukla’s (2021) non-ideal theory of sexual consent, we directed this study toward examining and disentangling a sexually diverse sample of young men’s discourses of alcohol use and sexual consent.

### Methodology

This qualitative study deploys discourse analysis as the overarching research design. Discourse analysis is a usefully deconstructive approach to examining social processes that (re)produce knowledge and power relations through systems and structures, which constitute how specific “issues” (e.g. alcohol, sex, and consent) are understood, experienced, and discussed (Fairclough, 2003). With discourse analysis, we sought to analyze the dynamics, contexts, and effects of how young men talk about consent, ethical sex, and alcohol use. This involved positioning the speakers (young men) as both affected by, and engaging in, the construction of normative meanings of (ethical or non-ethical) sex they are having when using alcohol. Thus, we attended not only to the narratives recounted to us by young men, but also to how these narratives may constitute taken-for-granted knowledges, and the transpiring of social actions and interactions (i.e. discourses) related to sexual consent in the context of alcohol use.

### Sampling and recruitment procedures

This study extends from a broader program of research exploring young men’s use of substances (including alcohol) with sex. A stratified purposive sampling strategy (Knight et al., 2017) was used to ensure the sample comprised wide-ranging participants within the criterion of young men, including men of diverse sexual and ethnocultural identities. We recruited participants by posting study advertisements over social media (Facebook, Instagram) and across clinical and community sites. These included a youth-driven non-profit organization, a men’s sexual health and primary care clinic, and two community-based research offices focusing on men’s sexual health and young people’s alcohol and other substance use. Recruitment materials were titled with the name of this study, “Sociocultural Contexts and Young Men’s Sexual Lives,” and indicated that researchers were seeking to interview young men about sexual health and substance use. The specific eligibility criteria were as follows: live within Metro Vancouver, fluent in English, self-identify as a man, between 15 and 30 years of age, currently or previously sexually active, and have used substances (any) with sex. Participants provided informed consent and received a CDN $30 honorarium. Ethics approval was obtained from the University of British Columbia Behavioural Research Ethics Board (##H16-01915-A013).

### Data collection

We conducted in-depth, semi-structured interviews with 76 young men. Individual interviews (Jan 2018–Feb 2021) ranged in length form 1 to 2 hours and took place at research offices in downtown Vancouver; 10 interviews were held remotely over Zoom following the onset of the COVID-19 pandemic. The interview guides were structured to elicit comprehensive discussion of participants’ perspectives, experiences, and contexts of using substances (inclusive of but not limited to alcohol) with sex. The primary interview question serving the current investigation was: “*How has substance use influenced your ability to negotiate the kinds of sex you are having?*”. This question included the follow-up probes: “*What does ‘consent’ in the context of sexual activity and/or sexual relationships mean to you?*”, and “*How ‘in control’ of your decisions around substances do you normally feel? How does this affect your ability to negotiate safer sex and/or consent?*”, alongside other interviewer-initiated prompts. Of note, the interviewers did not explicitly define key terminology used with these questions (e.g. “sexual activity,” “consent,” and “negotiate”), as we sought to have participants take the discussions in directions they wanted, drawing on their own language and understandings of sexual consent. Furthermore, we did not exclusively focus on alcohol with our questions, though, as the interviews unfolded, we noted that alcohol was the substance featuring most saliently in men’s discussions of consent and sexualized substance use. We therefore began using targeted probe, prompt, and loop questions (Oliffe and Mróz, 2005) to unpack how alcohol, specifically, ties into men’s discourses of sexual consent. Interviews were audio-recorded, transcribed verbatim, accuracy checked, anonymized, and labelled with participant-selected pseudonyms. Participants also completed a socio-demographic survey.

### Data analysis

The dataset was initially categorized using a preconfigured codebook, which our team developed by drawing on prior research experiences, engaging with the sexualized substance use literature, and using constant-comparison and open-coding techniques with the initial transcripts (Charmaz, 2014). Data immersion was facilitated by undertaking a related thematic analysis leveraging the same dataset (Goodyear et al., 2023). This study found that using alcohol before sex can influence young men’s perceived capacitates for navigating conversations about, and actions related to, consent in the context of sexualized alcohol use. Yet, we completed that analysis without unpacking the normative considerations young men raise (or do not raise) about their understandings of alcohol use and sexual consent amid evolving social contexts. Thus, for the current investigation, we revisited the dataset and focused on data coded as “sexualized use of substances,” “depressants” (including alcohol, toward which we focused our attention), and “sexual coercion and consent.” Our inquiry was advanced by pulling apart and analyzing data coded at “sex, gender, and sexual orientation” and “culture” (e.g. queer cultures and dating/hook-up cultures). The lead author iteratively grouped and collapsed different configurations of these codes whilst reviewing the corresponding data pulls. The coded data and full transcripts were further examined to develop preliminary insights about young men’s discourses of alcohol use and sexual consent. Data were managed using NVivo 12 and Microsoft Excel software.

We used standard practices of discourse analysis to enhance and transform our early insights, including analyzing both textual and contextual elements of the data, and interrogating explicit (e.g. self-disclosures and debates) and implicit (e.g. assumptions and values) threads of consent-related discourse (Fairclough, 2003). To develop overarching conceptual categories, we examined the meaning and social effects we (and participants) ascribed to the textual data while concurrently exploring interconnections between power relations, ethical considerations (e.g. with respect to autonomy and capacity), and young men’s contemporary understandings of consent. This involved us critically examining *what* young men construct as sexual consent in the context of alcohol use, *how* these understandings are constructed, and *why* these understandings may be constructed in particular ways (i.e. for what possible reasons, and with what implications). The analysis led us to identify several, often mixing and contesting discourses, which we classified into three organizing discourses that are henceforth referred to as “discursive frames.” These include: (i) *careful connections*: this discursive frame centered discourses of care, connection, and control in young men’s discussions of actively promoting sexual and decisional autonomy; (ii) *watering it down*: this discursive frame featured discourses of disinhibition, deflection, and denial to normalize alcohol use as being somewhat excusatory for sexual violence, downplaying the role and responsibility of men themselves; and (iii) *blurred lines*: this discursive frame included discourses of a continuum of consent and “meeting (masculine) expectations,” with young men describing a complicity with sustaining masculine discourses, amid contesting and conceding marginalities in talking through personal experiences and community norms for alcohol use and sexual consent. These discursive frames are presented below and substantiated with selected participant quotations, whose text, meaning, and social effects (Fairclough, 2003) we collectively interpret to provide a schematic picture of young men’s discourses of alcohol use and sexual consent.

## Findings

The 76 young men in this study were between the ages of 18 and 30 years (mean = 23.9). Of these, 71 identified as cisgender men and 5 as transgender men. Participants identified as gay (*n* = 33), bisexual (*n* = 19), pansexual (*n* = 5), queer (*n* = 4), and straight (*n* = 19). The sample was ethnoculturally diverse, with people identifying as white (*n* = 41), Indigenous (*n* = 11), Black (*n* = 7), and other racialized ethnocultural identities (*n* = 27). Participants could select multiple response options when asked their gender identity, sexual orientation, and ethnocultural identity. Of note, the sample included young men who used a range of substances with sex, including cannabis, poppers, crystal methamphetamine, MDMA (methylenedioxyphenethylamine), cocaine, GHB (gamma-hydroxybutyrate), and ketamine, among others. However, the present analysis focuses on young men’s discourses pertaining to sexual consent and alcohol, as this was the substance participants most widely used with sex and because we wanted to examine intersections between sexual consent and *alcohol* use, specifically.

### Careful connections: Discourses of care, connection, and control

In describing how alcohol featured in their sexual encounters, the young men were strongly influenced by a discursive frame of careful connections. The men invoked discourses of “care” and “connection” as they underscored their efforts to proactively consider and openly talk about dynamics of consent in the context of sex involving alcohol. Frequently, they described consuming small amounts of alcohol prior to having sex, with the aim of pre-establishing or strengthening a sense of connectedness. Moreover, having social drinks prior to sex allotted space for discussion of sexual preferences, histories, and boundaries, thereby helping to facilitate sexual safety. This was especially so for participants who wanted to cultivate conditions for care and connection in advance of having sex with someone for the first time. For example, Valentina shared his reasons for connecting over alcohol and cannabis before sex:

*Especially with random hookups, too. It’s, like, “Hey, let’s smoke this joint.” [. . .] Or, “Let’s have a couple glasses [of wine].” You know what I mean? It’s always a social thing. It gives that time. . . that whatever, 15, 20 minutes before you actually do the deed [i.e., have sex], or whatever. [. . .] Like, checking in to see where they are, and it gives you that time. [. . .] It gives you an opportunity just to see how their day’s going and kind of just, not suss them out as a person, but it totally, it gives you an opportunity to check in on them. [. . .] Instead of just, like, going and saying hi and sticking your fucking penis in their ass. [. . .] You want to see what you’re about to get into. And it relates back to trust and whatever, going on beforehand and you want to see what’s about to happen (Valentina, a 29-year-old gay man).*

As above, in contrast to dominant discourses of masculinity and men’s sexuality—which can emphasize stoicism and libido—participants positioned themselves as assessing and deciding consent through some level of relational connection with new partners. This self-construction seemed to be especially prominent among—but not restricted to—the young GBTQ men we interviewed. These participants tended to invoke the discursive frame of careful connections when describing how alcohol can serve as a mechanism to connect and disinhibit, often in rather calculated ways, such as to buy some time to scaffold the conditions for ethical consent to occur.

Other key aspects of this discursive frame were men’s concerns about how their capacity to have command over situations when using alcohol too heavily could impair capacities for responsible sexual decision-making, notably in circumstances in which they or their sexual partner(s) may be especially vulnerable. In tension with expectations of normative masculinity and young men’s “risk-taking,” a discourse of “control” reflects how participants tended to take great care when actively choosing to use alcohol with sex. Evident was men’s desire to feel relaxed but cognisant and with some level of control in their sexual encounters, making informed risk assessments as they first connected with sexual partners. For example, in the context of having drinks prior to sex, Manuel, a 19-year-old gay man, shared that he will take the time to consider “*how sane the person looks*,” “*the contact or the way they communicate with me*,” and “*what their [perceived] intentions are*.” These sorts of social and relational judgments in turn affect the steps he will take to “*try to watch out for [him]self*,” including by slowing down or limiting his use of alcohol to guard against being taken advantage of. Participants underscored the temporal dimensions of consent as they described being wary about threats to sexual safety that can arise when one sexual partner is more intoxicated than the other, especially when in unfamiliar settings or with new partners. In these encounters, GBTQ participants in particular took up a discourse of control when expressing concern about being coerced by men who they perceived to have lost restraint and accountability due to alcohol use. For example, Mark shared:

*I just take each situation as it comes. I’ve definitely been in a situation where I met someone online, went to meet up with them, and they were just belligerently drunk. And I’m like, “Nope, fuck ‘em.” I got out of there, you know? Because clearly that was not a good place for me to be and, clearly, they were not in a place where they could be making decisions. I mean, maybe that is what they do every day so I can’t – who am I to judge that. And who am I to say they weren’t in control. But, from my perspective that was just not a good thing. And so, I didn’t want to be a part of that. [. . .] Not just for the selfish reason that it could come back to haunt me but, I mean, that I think is – is one of the biggest risk factors there, for me. But also, it’s – who knows what they’ve been going through that day and maybe they’ll regret it in the morning and maybe, you know, you’re making the right choice for them. But again, who are you to choose for other people? So, it’s very delicate. Very dangerous (Mark, a 29-year-old man who identifies as homoflexible).*

As Mark illustrates, the discursive frame of careful connections reflects the care for self and others that men describe having when alcohol is involved with sex. Participants indicated that heavy intoxication could tilt sexual risk levels and power dynamics toward the unacceptable whilst obscuring capacity for meaningful connection, at times outweighing the desire for pleasure and physical release. In turn, they surfaced the need to establish careful and caring connections that were frequently buttressed by notions of masculine responsibility of caring for both self and others. Yet, this motivation to care for self and others occasionally came in conflict as men strove to avoid coming across as paternalistic or controlling of their sexual partners’ choices. This can be seen in Mark’s self-reflective comment, “*Who are you to choose for other people?*”, which seems to reflect a desire to promote the autonomy of others whilst still working to retain and uplift one’s own decisional capacity, including by withdrawing consent as a form of mutual protection (i.e. “*You’re making the right choice for them.*”).

Young men leveraged the discursive frame of careful connections when characterizing their (often cautious) lead-up to sex involving alcohol, including as they recognized and worked to mitigate the possibility of sexual consent being breached. Indeed, although using alcohol in small amounts could facilitate care, connection, and control with sex, doing so could also function as a double-edged sword of sorts, most notably in contexts of heavy drinking or disparate levels of intoxication. Here, participants seemed keenly aware of the risk for getting lost in the moment and for “lines to be crossed” amid sexualized alcohol use, thereby prompting them to pre-emptively look out for their own sexual safety and that of their sexual partners. The criticality of careful connections when drinking was emphasized by Finch, a 24-year-old straight man:

*Alcohol is a prime example of that, where you’re not really thinking about the consequences of your actions. It’s a really tough dialogue to have with people if you’re in that [intoxicated] headspace, so. [. . .] In the context of, like, are both people consenting? Are they enjoying themselves? Was it what they intended? That’s never really been an issue for me, I’m pretty sure. And usually there’s a pretty extensive dialogue, or a foundation to the relationship that exists before I have sex with someone, so I never feel like I’ve ever crossed that line of consent. It’s usually been like really clear (Finch).*

At times, the young men invoked an ideal of “intoxication parity” (Hunt et al., 2021) as one prominent strategy to avoid sexual encounters that could later be characterized as having lacked enthusiastic, genuine consent. With this approach, participants emphasized that consensual sex is best achieved when all parties have consumed only small amounts of alcohol (or none whatsoever) and are still “in control,” or when sexual partners are equally intoxicated and ostensibly operating with the same degree of control. This discourse, and men’s desire to promote more equal power relations and conditions for fair play, featured in both casual sexual encounters and established relationships, though with seemingly more leeway in the latter context. For instance, Byron shared:

*If it’s my partner, it’s like pretty much like all the time [that we’re drinking with sex], but I don’t know. I kind of struggle with that, actually. Like, what if like one of us is drunker than the other one? Like, that’s happened before, and it seemed good, but part of me was like, “Was that okay?” [laughs uncomfortably]. Although, you know, I know that if I told her, she would be like, “Yeah, we’re together, [so it’s okay]” But I don’t know. Like, if I couldn’t understand what the other person was saying, I’d be like, “No way, Jose.” Or if I was sober, or if I also wasn’t drunk, then yeah, for sure. (Byron, a 22-year-old man who identifies as bisexual and pansexual)*

By centering discourses of care, connection, and control in contexts of sexualized alcohol use, the young men actively worked to promote sexual and decisional autonomy as they looked out for themselves and their sex partners. Yet, as our analysis progressed, it became clear that there were also potentially autonomy-compromising situations involving sex and alcohol.

### Watering it down: Discourses of disinhibition, deflection, and denial

Participants’ perceptions and experiences of violated consent in the context of sex involving alcohol were frequently embedded within a discursive frame of “watering it down.” This frame involved men normalizing alcohol use as the primary cause of sexual coercion and assault, while concomitantly deflecting and denying the role and responsibility of men. In this context, participants deployed discourses of deflection to emphasize how the disinhibiting effects of alcohol could compromise men’s decisional autonomy, with several emphasizing how alcohol could directly disrupt their self-control when it came to opportunities for having sex with impaired partners. Evident in the quote from Finch below, sexual assault can be framed as a direct consequence of (women) drinking too much. In this scenario, the unequal gendered dynamics of sexual assault risk are acknowledged yet subsequently denied:

*Obviously, there’s the scenario of, like, you drink too much. Specifically, within the context of girls, like they might drink too much, and then they’re so inebriated that a guy, like, takes them home or something like that, or sexually assaults them without being aware. That’s obviously a consequence (Finch, a 24-year-old straight man).*

Finch positioned himself and heterosexual men more generally as somewhat disinhibited to the point of being “unaware” of the potential consequences for their actions when too much alcohol was involved. Aligning with dominant discourses of masculinity prizing sexual conquest as an ideal of a drunken night out, there was a disregard for the woman’s agency and denial of the man’s culpability for not ensuring that, thereby reflecting, and reinforcing heteronormative misogyny. Indeed, Finch and others actively deflected notions of violation by conflating alcohol (over)use and sexual consent for both parties, emphasizing instead that risk resides with men’s partner(s). This deflection of responsibility was even more pronounced as men explicitly spoke of alcohol use, sexual assault, and issues of power and consequence. For example, Brazil claimed “expertise” regarding the gendered power dimensions that are animated in the context of men’s alcohol overuse, whilst discursively separating himself from men’s predatory practices. In doing so, he emphatically described alcohol as a “*rapey drug*”:

*With alcohol, you can get rapey. That’s the bottom line. [. . .] You can lose control. Like, I will lose control over my mindset. It’s not like I’m out raping people. And it’s not like people who get drunk are out raping people, [. . .], but alcohol is, like, your decision-making skills are considerably reduced. [. . .] I see alcohol as a rapey drug. [. . .] And this is a dude saying this, who’s in a dominant position who also fucks women. So, I actively think about these things. Lots of people don’t, and that’s the problem. Alcohol, even if you get too fucked up, that’s just when you start to get to the rapey aspect (Brazil, a 19-year-old man who identifies as straight and pansexual).*

The men constructed alcohol as a potentiator for sexual violence to occur, regardless of who (aggressor, victim, or both) was intoxicated. Pertinent examples surfacing across our interviews included experiences of hazing, sexual humiliation, and sexual entitlement. For example, Lorne, a 24-year-old man who identified as gay and trending toward asexual, shared an experience of having witnessed multiple intoxicated people sexually assault an unconscious, severely intoxicated man at a bathhouse. While this participant understood and acknowledged that performing sexual acts with an unconscious person is unacceptable (because it is rape), he seemingly deflected the wrongness of this event by attributing it to alcohol intoxication, commenting: “*Because your judgment is gone out the window, you know, you can’t think rationally; people were coming in doing things, whatever they wanted [to the unconscious man].*” Another example of deflecting the shared role and denying responsibility of men in sexual violence amid alcohol intoxication was illustrated by Lee, a college resident advisor, as he described a group sexual assault that took place:

*I get a lot of sexual assault disclosures. So, I think one that stands out to me recently is like a student got assaulted at a party. A male student got assaulted by another male student. Yeah, it was just a lot of touching, and he put his dick on the other guy’s butt, and then he was just. . . It was a group of them, and it was the whole [group]. But I know they were all drinking, so. Not that, not the survivor in that instance, but like the like perpetrators, so. . . (Lee, a 22-year-old pansexual man).*

Lee discursively reveals an understanding of the sexual assault that had occurred, even using the language of “survivor” to refer to the victim, while successively watering down the impact of the event as men “being men” and using alcohol (“*But I know they were all drinking, so*. . .”).

Evidence of young men deflecting the responsibility of sexual assaults due to alcohol use also featured within the interviews of those who shared with us that they had previously experienced sexual violation. For example, Sandy framed his personal and peer experiences of sexual violation as the direct “result” of having consumed alcohol. Concerningly, he goes so far as to position himself and his friend and partner as “lucky” for each having “only” been sexually assaulted once, given their heavy alcohol use at that time of their lives. This experience ultimately led him to cease or at least reduce his alcohol use:

*I’ve been sexually assaulted while drunk. [. . .] My best friend’s been sexually assaulted, and roofied as a result of alcohol. And I think my partner’s been sexually assaulted as a result of alcohol. [. . .] Each of the three of us have been lucky enough that we’ve only been sexually assaulted once, and it was like in the part of – or like at least for me – it was in the part of my life where I’d only ever used alcohol [and no other substances] (Sandy, a 30-year-old queer man).*

Participants sometimes constructed themselves as “at risk” and more vulnerable to sexual violation while intoxicated. To this point, Ryley, a 19-year-old gay man, cautioned that being intoxicated “*leaves me susceptible to god knows what*.” This perception was especially, though not exclusively, common among the GBTQ participants. GBTQ men and some others described diverse experiences with sexual violation, ranging from guilt and shame about taking part in sexual encounters they (in hindsight) may not have consented to sober, to more explicit occurrences of sexual coercion and assault, such as men’s perceived aggression and the gendered and physical power dynamics described by Connor:

*I think at least for me that mostly revolves around alcohol. Guys can become too aggressive, and then, you know, women or sometimes smaller guys can become vulnerable to more aggressive individuals. Or even themselves, when they’re just too drunk (Connor, a 20-yeard-old gay man)*

Notwithstanding that alcohol and violence can intersect in complex ways, the discursive frame of “watering it down” served to background the role of men and masculinity in experiences of sexual violence. Yet, some began to critically interrogate how this discursive framing could deflect notions of responsibilization away from the sexual perpetrator; for example, Jacob, a 22-year-old straight man, reflected, “*you kind of blame it on the alcohol, but, looking back on it, you should blame the person*.”

### Blurred lines: Discourses of a continuum of consent and “meeting (masculine) expectations”

The discursive frame of “blurred lines” emerged as young men discussed a continuum of sexual consent, including the legitimacy of (violated) consent in the context of sex involving alcohol. Evident was participants “meeting (masculine) expectations” when talking about personal experiences and community norms of sexual violation while intoxicated, and of having followed through with sexual encounters they subsequently came to regret. These discourses manifested as the young men recounted instances in which they had (drunkenly) taken part in sexual encounters where there had been low levels of interest, yet where they proceeded anyway due to perceptions of pressure and expectation from their sexual partner(s), peers, and/or themselves. When looking back on these experiences and the discord between men’s feelings and actions, participants sometimes second-guessed whether they had even wanted the sexual encounters to occur. The ways in which they deployed these discourses suggested that, at times, following masculine discourses that prize sexual desire and performance led these young men into sex they had not been enthusiastic about—or had outright declined. Indeed, this “pressure” young men described facing when deciding whether to have sex seemed tied up in broader masculine norms, with men’s pursuit of sexual prowess shaping their overall decision-making and, in some cases, their susceptibility to sexual coercion and assault. For example, Brazil (introduced above) questioned the appropriateness of sexual activity he had participated in drunk, whilst concurrently implying that he felt he had an onus to follow through on the sexual activity:

*I didn’t even really want to do it that much, but I was drunk, and I was in their bed, and I was, like, “Ah, well, I’ll start it. Fuck it.” In hindsight, I probably didn’t want to do it but I did start it because I felt like it was gonna happen anyway. And then I really got into evaluating whether or not I actually wanted to do it, or whether I felt comfortable. And I was, like, well, I did start it but, uh. . . I get into that whole thing. [. . .]. It just wasn’t a good idea. Whether or not I wanted it, it was kinda hard to tell ‘cause I was drunk. I feel like if I was sober enough, I would’ve just went home, but, you know (Brazil, a 19-year-old man who identifies as straight and pansexual).*

A salient feature of the continuum of consent discourse is how young men muddied the waters of what sexual consent *is* and what should (and should not) be considered violated consent. This seemed to be a discursive strategy through which young men rationalized the ways in which their expectations and decisions related to consent in the context of sex involving alcohol took shape in the moment, rather spontaneously. This was especially so for sexual encounters involving alcohol and other substances, about which participants stressed that the incapacitation that can come with severe intoxication could inhibit true consent, regardless of the pleasure they and their partners may have experienced in the moment. At times, the young men did not explicitly identify themselves as victims of sexual assault—arguably a normative frame for men—but nonetheless recognized having been marginalized and coerced by other men. For example, Tracao described being pressured into taking alcohol and GHB (another depressant) by a sexual partner and enjoying himself at the time but later coming to regret the encounter, once sober:

*I wasn’t completely out [of the closet] and I was sort of just not completely there [mentally, because I was drinking], as well. So, I was enjoying [laughs]. . . I enjoyed it, from what I remember. And when I came to, that’s when it was more of a big shock. Like, I can’t believe he did that (Tracao, a 23-yeard-old bisexual man)!*

Participants’ views on the continuum of sexual consent and violation varied widely. Both cisgender, heterosexual men and GBTQ men recounted experiences of sexual violation, many of which were downplayed or dismissed (whether by themselves or others) when disclosed. Graham, a 22-year-old gay man, detailed an incident of “stealthing” wherein his sexual partner removed the condom Graham was wearing, without his knowledge or awareness (which he described as being altered because he had been intoxicated). Once Graham discovered that the condom had been removed, he and his partner disagreed on whether doing so had been appropriate, leaving Graham to reflect “*it became a whole thing [i.e., issue]*” in their relationship. Sandy and his partner had similar experiences with what he labelled “*the spectrum of sexual assault*” that young men risk exposure to when drinking with sex, emergent from which are the temporal dimensions of sexual consent and shifting norms for what might be understood and spoken about regarding assault:

*My partner’s sexual assault and my sexual assault, it was less explicitly, like, sexual assault sexual assault. More like, grey sexual assault, where it’s like someone fucks us without a condom despite the fact that we did not consent to that 100 percent. [. . .] None of us evaluated that we were sexually assaulted until like five years, or eight years after the fact. And we were like, “Oh, that’s a type of sexual assault? Holy shit, I was sexually assaulted! Holy shit. I lost my virginity being sexually assaulted by a man” (Sandy, a 30-year-old queer man).*

As above, young men took up—and occasionally contested—the discursive frame of blurred lines wherein there was no objective consensus of what constitutes (or does not constitute) violated consent. This sense of a lack of clarity was evident in the perspective of Lorne, who, in recounting a sexual assault in his peer network, commented, “*Consent is a very fickle and fleeting phenomenon, especially in [the] gay community*.” He suggests the issue of blurred lines is especially prevalent in gay/queer communities, about which some GBTQ participants endorsed having or witnessing amongst peers a lower normative threshold for what is considered violated sexual consent. Intra-community dynamics aside, we note that experiences of violated consent and blurred perceptions of consent featured across the sample, including among cisgender, heterosexual men. For example, Peter shared that the boundaries of sexual consent are more easily bent and crossed when intoxicated; of note, he seems to contextualize this tension with the discursive drive to meet (masculine) expectations during sex:

*If I’m drunk and I’m not down for a girl, I’ll probably still fuck her anyways, especially if she’s like pushy and stuff. 100 percent. Like, buddy, one time I was drunk in my bed after a party, and this chick literally comes into my room, and she’s like, “Hey, do you want to like – do you want to fuck?” And I’m like, [name], no. I’m not down.” She’s like, “Come on. I’ll suck your dick.” I’m like, “No, not down.” Four or five times. And then she starts rubbing my dick, sucks me off, fucks me, and then stays the night. I’m like, argh! Dude, honestly, if I was sober, I’d be like, “Get the fuck out.” I would not have been down. Oh, but [when] I’m drunk, like, “Okay, fine. Whatever.” [. . .] I definitely find like it’s easier to cross a line when you’re intoxicated (Peter, a 26-year-old straight man).*

Although complicit in sustaining a masculinity that prizes sexual conquest, Peter simultaneously concedes having been marginalized and discursively positions alcohol as the excusatory factor for giving into that marginality. Yet, even when the young men explicitly recognized that consent had been violated amid sexualized alcohol use, they tended to downplay the seriousness of these encounters. Doing so involved men employing the blurred lines discursive frame whereby they talked up the role of alcohol and related influences (e.g. peer pressure and partying) in shaping these sexual encounters, seemingly to draw focus away from the violation itself and to avoid being framed as a victim. These processes of deflection were caught up in hegemonic masculinity and norms around male (hyper)sexuality. Indeed, Drake, a 20-year-old bisexual man, shared that young men’s sexual scripts—what he called “*this very strange form of banter that happens [about men and sex]*” —have created a context in which men are expected to be sexually assertive, leading many to be “*imitating what they think being a macho guy is*” when negotiating and having sex. This “guy talk” also featured the minimization of victimhood following sexual assault—an issue that featured quite prominently across the sample and categories of sexual identity. To illustrate, Kim downplayed an experience of sexual violation by “shrugging off” its seriousness (“*that was kind of funny, but it wasn’t funny*”). In this way, he seemed to blame alcohol and “*bad life choices*” to rationalize what happened while preserving his (hypermasculine) sexuality, and later removing alcohol from his life:

*If you drink too much, you can’t really think or actually give consent. There was one time, when I was in high school, I didn’t give consent, but this chick just tried to go to town. But I was lucky [laughs]. Another friend, like, grabbed me while my pants were around my ankles. [He was] like, “No, no, no,” and then dragged [me] out butt naked. That was kind of funny – but it wasn’t funny, at the same time, looking back on it. I guess she tried to take advantage of me. [. . .] That’s why I don’t really drink much anymore. You just make bad life choices. [. . .] I just thought she was thirsty [i.e., eager for sexual attention]. [laughter] Looking back on it. [. . .] I guess the fact that I didn’t really know what was going on, which I attribute back to alcohol, is why I don’t really do it that much anymore (Kim, a 26-year-old straight man).*

Participants’ ambiguity about consent in contexts of sexualized alcohol use was heavily embedded in the discursive frame of blurred lines. Here, the discourse of a continuum of consent manifested as men rationalized the “messiness” of consent amid sexualized alcohol use. This even occurred as men recounted past sexual violations, the legitimacy of which they sometimes downplayed by talking up the role of alcohol in shaping these encounters whilst concurrently uplifting a discourse of “meeting (masculine) expectations” during sex.

## Discussion

This study examines young men’s discourses of alcohol use and sexual consent to identify the dynamics, contexts, and practical effects of those experiences. Participants centered a discursive frame of careful connections when discussing their efforts to actively promote sexual and decisional autonomy as they look out for themselves and their sexual partners when drinking. Conversely, they invoked discourses of disinhibition, deflection, and denial to normalize alcohol use as the primary cause of sexual violence and downplay the role and responsibility of men. Finally, the young men operationalized discourses of a continuum of consent and of “meeting (masculine) expectations” when talking about personal experiences and community norms for alcohol use and sexual consent, including as they discussed sexual violence and victimization while intoxicated. These findings shed light on young men’s discursive understandings of sexual consent in the context of alcohol use, highlighting how young men construct and contest the kinds of (ethical or non-ethical) sex they are having under non-ideal and potentially autonomy-compromising conditions.

Findings from this study and specifically the discursive frame of *careful connections* offer insight into how non-ideal consent models are being taken up in the contemporary contexts of young men’s lives. The interviewed young men detailed their understandings and relations of consensual sex while intoxicated, including how they negotiate sexual and decisional autonomy. Extending upon pertinent literature on key elements attributed to sexual consent (Beres, 2007; Kukla, 2021; Muehlenhard et al., 2016), this study underscores that dynamics of consent are socially constructed and heavily contingent on context, notably with respect to young men’s evolving gender norms and sexual scripts. Even when the young men we interviewed described instances of them and/or their sexual partners being intoxicated, sometimes heavily, they indicated that consensual sex was still possible. This “real-world” example of non-ideal sexual consent has pragmatic implications for sexual health praxis, particularly given that achieving enthusiastic consent—while certainly desirable—is not always possible. Following Kukla (2021: 271), we assert that “compromises to our autonomy are the norm, not the exception.” Sex involving alcohol is one potentially autonomy-compromising sexual encounter, though there are myriad other factors that can compromise autonomy during sex, such as trauma, dementia, exhaustion, and some kinds of disability, as well as gendered and other power imbalances (Kukla, 2021). However, there are likewise ways to mitigate compromises to autonomy, as is evidenced by the current study’s findings about how young men—especially GBTQ men—take up and integrate the discursive frame of careful connections. Appreciating the reality and familiarly of many autonomy-compromising situations, it would be prudent for future work to continue examining how well-scaffolded, ethical, and consensual sex can occur under non-ideal conditions. This is not to disparage or circumvent enthusiastic consent models, but to advance more realistic understandings of how consent can still work “well enough” when autonomy becomes (partially) compromised, as our findings show is a commonplace occurrence.

This study’s analysis of the *blurred lines* discursive frame adds to our understandings of how young men conceptualize and deploy continuums of consent and intoxication. Consistent with recent work (Carline et al., 2017; Hunt et al., 2021; Whittington, 2021), the young men we interviewed described sexual consent along a continuum that captures more extreme examples of sexual violation to more positive and agentic encounters, including those involving alcohol as a way to promote care and connection. Continuum thinking with sexual consent can encourage people to consider more carefully the processes and effects of sexual negotiation, and to reflect on how these issues are influenced by aspects of context, such as gendered or physical power dynamics (Whittington, 2021). This may be especially important in the context of sex while intoxicated, where, in line with work involving heterosexual young men and women (Hunt et al., 2021), issues of sexual consent and acceptable (and unacceptable) sexual activities can become increasingly blurred. Perhaps distinct from Hunt et al.’s (2021) study, in which “blurred lines” largely occurred amid moderate (vs mild or heavy) intoxication, the men in our study also invoked the discursive frame of “blurred lines” when recounting sexual encounters wherein one participant had been clearly and heavily intoxicated. This disparate finding may be attributable to our study’s sampling frame of young (mostly GBTQ) men, who face unique consent challenges prior to, during, and in response to sexual encounters. This is due, in part, to the ways in which cisheterosexism has reinforced the hiding, denial, and shaming of GBTQ men’s sexual practices (Gaspar et al., 2021; Richardson, 2022). Still, in line with research showing that GBTQ men leveraged masculine responsibility and assertiveness to avoid sexual coercion amid #MeToo (Gaspar et al., 2021), we found unique forms of agency and control in how young GBTQ and other men are using *careful connections* to foster ethical and consensual sexual encounters.

Young men in this study occasionally expressed confusion and unease when invoking the discursive frame of “blurred lines,” including as they recounted experiencing sexual victimization while intoxicated yet of having felt unsure—in the moment or in hindsight—whether sexual consent had truly been violated. This finding extends upon prior research indicating that most men who have experienced sexual victimization do not label this as sexual abuse or rape (Artime et al., 2014), as well as more recent work suggesting that some GBTQ men are questioning the acceptability of sexual practices they may have once passed as “acceptable” before #MeToo and shifting community norms and boundaries around sexual consent (Gaspar et al., 2021). Conflicting feelings and difficult conceptualizations of sexual consent and sexual assault are well documented and have even been theorized in connection with gender role stereotypes about masculine invulnerability and sexual insatiability, which contribute to the under-reporting, late reporting, and limited help-seeking among men who experience sexual violence (Artime et al., 2014; Petersson and Plantin, 2019; Weiss, 2010a, 2010b). This under-reporting and conventional masculinity norms also intersect with commonly held myths about “male rape” (Artime et al., 2014; Boyle and Rogers, 2020), such as “it is impossible. . . to rape a man” and “men [who are raped] are to blame for not escaping” (Struckman-Johnson and Struckman-Johnson, 1992: 90), with these myths discursively positioning men as strong and always in the mood for sex. The present study provides related insights into young men’s drive to meet (masculine) expectations and their perceptions of sexual victimization while intoxicated, though continued research is needed to explore these dynamics and men’s evolving understandings and acknowledgment of sexual assault and victimhood.

Findings on the discursive frame of *watering it down* hold promise for informing interventions focused on the nexus of men, masculinities, alcohol, and sexual violence. This is significant insofar as this nexus is currently under-addressed in alcohol policy, with one study arguing that the role of gender and specifically men here tends to be “noticed and then forgotten” (Farrugia et al., 2022: 1422). Similar to Farrugia et al.’s (2022) study, the young men we interviewed backgrounded the role and responsibility of men in experiences of sexual violence in the context of alcohol use, oftentimes attributing sexual violence and aggression to the substance rather than men’s own actions. Young men’s narratives here can be interpreted as potential moments of contestation and disruption vis-à-vis the extent to which they envision sexual consent and violation, including in the evolving context of #MeToo, and are important targets for efforts addressing men’s role in sexual violence (Carline et al., 2017). Others have underscored opportunities for socio-ecological interventions that concentrate upon men (and masculinities) whilst not over-individualizing the causes of sexual violence, including by accounting for the wider exo-, meso-, and macro-level influences shaping men’s experiences of sexual consent and violation (Artime et al., 2014; Carline et al., 2017; Hunt et al., 2021). Research on the continua of consent has also called for policy and practice responses that leverage an expanded model of consent and that focus on and nuance the ethics and contexts of young people’s sexual encounters, “rather than on a heavy-handed legal binary of consent/rape” (Whittington, 2021: 494). Still, much room remains for targeted research and interventions that address the ways in which sexualized alcohol use intersects with young men’s evolving societal contexts.

This study has strengths and limitations. The large, sexually diverse sample and study design focused on prevailing findings across the sample rather than differences between subgroups of men. The limitation herewith is that temporal changes and within-sample differences across young men’s experiences cannot be fully determined. This study being set in Canada also limits the reach and representative of the findings to locales grappling with similar challenges around alcohol use and sexual consent. Longitudinal, multi-locale, and intersectional studies could further illuminate the gendered dimensions of young men’s alcohol use and sexual consent.

## Conclusion

This study offers empirical and normative insights into GBTQ and cisgender, heterosexual young men’s discourses of alcohol use and sexual consent. Findings on discourses of care, connection, and control underscore the ways in which young men use small(er) quantities of alcohol to foster ethical and consensual sexual encounters. Meanwhile, findings on discourses of disinhibition, deflection, and denial capture young men’s backgrounding of their own role and responsibility vis-à-vis sexual violence, directing attention instead to alcohol’s influences. Finally, discourses of a continuum of consent and of “meeting (masculine) expectations” reflect how young men understand and are affected by personal and community experiences of sexual violation. Taken together, these discourses provide insights into the gendered nature of sexual violence and the extent to which idealized notions of sexual consent play out in the everyday lives of young men who use alcohol with sex. These findings underscore the ways in which discourses of masculinity can enrol men in sexual violence, as both offenders and victims, and carry important philosophical and pragmatic implications for contemporary efforts to scaffold sexual consent, including in non-ideal contexts such as sex involving alcohol.
